# Correction: Xu et al. Temporal and Spatial Dynamics of Tumor–Host Microbiota in Breast Cancer Progression. *Microorganisms* 2025, *13*, 1632

**DOI:** 10.3390/microorganisms13112593

**Published:** 2025-11-14

**Authors:** Qi Xu, Aikun Fu, Nan Wang, Zhizhen Zhang

**Affiliations:** 1 Ocean College, Zhejiang University, Zhoushan 316021, China; 3160102619@zju.edu.cn (Q.X.); n_wang@zju.edu.cn (N.W.); 2 Sichuan Clinical Research Center for Medical Imaging, Dazhou 635000, China

There was an error in the original publication [1]. The original data presented mean and standard deviation (Mean ± SD) and needs to be replaced with Linear Discriminant Analysis (LDA) scores and the corresponding statistical significance (*p*–values). The original figure captions a and b in Figure 6 were mismatched with corresponding images and need to be matched to images b and a, respectively.

The correction has been made to Results Section, 3.4 The Differential Taxa Analysis During Breast Tumor Growth, Paragraph 1 and the caption of Figure 6:

LEfSe analysis (LDA score > 4, *p* < 0.05) delineated temporally resolved microbial signatures across four tissue compartments during breast tumor progression. In breast tumors, hierarchical taxonomic enrichment progressed from Proteobacteria phylum (LDA = 5.41, *p* = 0.04), genus Sphingomonas (LDA = 5.40, *p* = 0.01), family Sphingomonadaceae (LDA = 5.40, *p* = 0.01), order Sphingomomadales (LDA = 5.40, *p* = 0.01), and class Alphaproteobacterial (LDA = 5.39, *p* = 0.03) dominance at week 3 to Actinobacteria phylum (LDA = 4.94, *p* = 0.03) prominence at week 5, ultimately shifting to Bacilli-class (LDA = 5.46, *p* = 0.01), species *Bacillus halodurans* (LDA = 5.09, *p* = 0.04), species *Streptococcus azizii* (LDA = 5.04, *p* = 0.005), species *Bacillus litoralis* (LDA = 4.68, *p* = 0.005), and species *Paenibacillus humicus* (LDA = 4.17, *p* = 0.01) dominance at week 7 (Figure 6a). Normal breast tissue mirrored tumor-associated patterns at weeks 3–5 but diverged at week 7, retaining only *Streptococcus azizii* (LDA = 4.01, *p* = 0.02) and *Paenibacillus humicus* (LDA = 4.44, *p* = 0.04) as significantly enriched species (Figure 6b). Spleen tissue exhibited distinct phasic dynamics with Firmicutes phylum enrichment (LDA = 5.37, *p* = 0.03) at week 3, followed by transitional equilibrium at week 5 (Firmicutes 45.51%, Proteobacteria 45.90%) (Table S1). By week 7, microbial reorganization emerged, featuring dual enrichment patterns—Proteobacteria (LDA = 5.24, *p* = 0.03), class Gamma-proteobacteria (LDA = 4.04, *p* = 0.01), order Cardiobacteriales (LDA = 4.27, *p* = 0.03), genus Ignatzschineria (LDA = 4.27, *p* = 0.03), Actinobacteria (LDA = 4.44, *p* = 0.04), and Corynebacteriales order (LDA = 4.05, *p* = 0.01)—coexisting with Sporosarcina genus (LDA = 4.30, *p* = 0.03) predominance (Figure 6c). Cecal content microbiota demonstrated progressive ecological succession (Figure 6d). Initial week 3 profiles demonstrated significant enrichment of Actinobacteria-derived lineages, specifically order Propionibacteriales (LDA = 4.11, *p* = 0.03) and family Nocardioidaceae (LDA = 4.13, *p* = 0.03). By week 5, microbial dominance shifted to Proteobacteria (LDA = 5.16, *p* = 0.02), class Delta-proteobacteria (LDA = 5.16, *p* = 0.02), and Clostridia (LDA = 4.53, *p* = 0.02) associated taxa, evidenced by enriched-order Desulfovibrionales (LDA = 5.16, *p* = 0.02), family Desulfovibrionaceae (LDA = 5.16, *p* = 0.02), and Clostridiales order (LDA = 4.54, *p* = 0.03), family Lachnospiraceae (LDA = 4.38, *p* = 0.01), genus *Clostridium* sp. (LDA = 4.22, *p* = 0.01). Week 7 marked Firmicutes (LDA = 5.27, *p* = 0.02) predominance through Lactobacillales order (LDA = 5.33, *p* = 0.02) expansion family Lactobacillaceae (LDA = 5.34, *p* = 0.02), and Lactobacillus genus (LDA = 5.34, *p* = 0.02). These temporal transitions establish distinct microbial ecologies across four tissues during breast oncogenesis.

**Figure 6:**
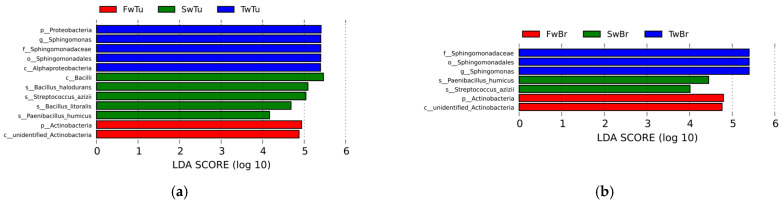

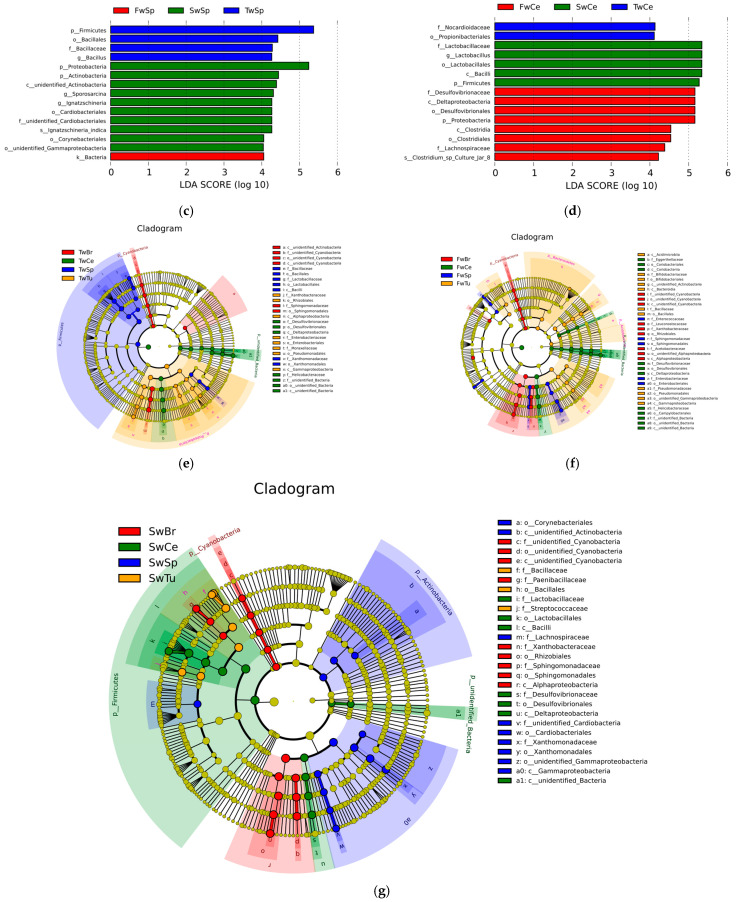
The differential microbial taxa in four tissues at different stages of breast tumor by LEfSe with the Tukey test (LDA score > 4, *p* < 0.05) and cladogram. (**a**) LDA value distribution in breast tumor at different stages. (**b**) LDA value distribution in normal breast tissue at different stages. (**c**) LDA value distribution in the spleen tissue at different stages. (**d**) LDA value distribution in cecal contents at different stages. (**e**) Cladogram in different tissues at 3 w. (**f**) Cladogram in different tissues at 5 w. (**g**) Cladogram in different tissues at 7 w.

The authors state that the scientific conclusions are unaffected. This correction was approved by the Academic Editor. The original publication has also been updated.
