# Supplementary material for: Temporal and Spatial Dynamics of Tumor–Host Microbiota in Breast Cancer Progression
Source: Microorganisms. 2025 Jul 10;13(7):1632. doi: 10.3390/microorganisms13071632 (PMC12300001; doi:10.3390/microorganisms13071632)
Supplement: Supplementary file 1 [file microorganisms-13-01632-s001.zip › Figure S2 The rarefaction curves of samples.pdf]

Supplemental materials

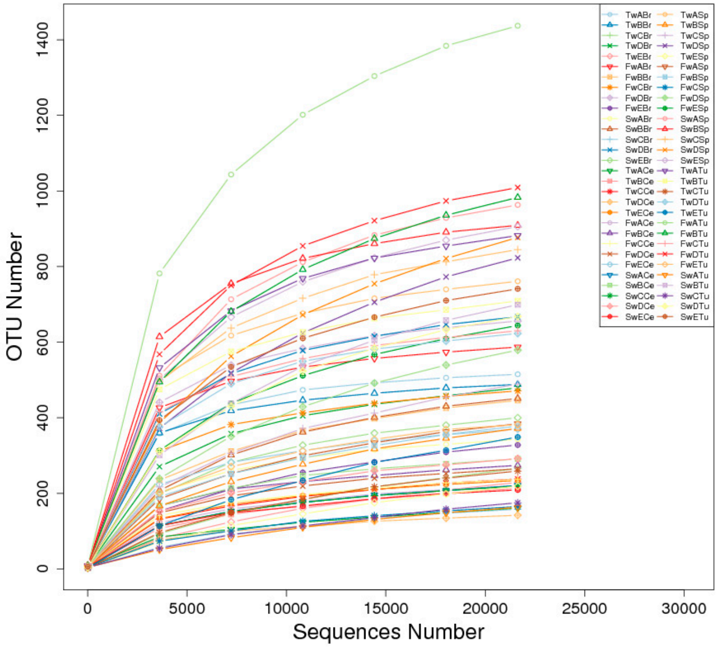

Figure S2. The rarefaction curve of samples.

Note: Tu, Br, Sp, Ce mean breast tumor, normal breast tissue, spleen tissue and cecal contents respetively; Tw, Fw, Sw mean 3 weeks, 5 weeks and 7 weeks respectively.
