# Supplementary material for: Temporal and Spatial Dynamics of Tumor–Host Microbiota in Breast Cancer Progression
Source: Microorganisms. 2025 Jul 10;13(7):1632. doi: 10.3390/microorganisms13071632 (PMC12300001; doi:10.3390/microorganisms13071632)
Supplement: Supplementary file 1 [file microorganisms-13-01632-s001.zip › Table S1. The abundance of microbiota at the phylum level in four tissues during tumor progression. docx.pdf]

Table S1. The abundance of microbiome at the phylum level in four tissues (n=5, %)

groups	Firmicutes	Proteobacteria	Actinobacteria	Bacteroidetes	Cyanobacteria	Acidobacteria	Chloroflexi	Fusobacteria	Spirochaetes
TwBr	17.18±2.42	70.05±5.75	3.06±0.04	2.74±0.07	2.55±0.18	0.14±0.00	0.11±0.00	0.18±0.00	0.00±0.00
FwBr	29.83±6.05	38.81±6.54	13.13±4.6	6.95±0.74	4.37±0.12	0.58±0.00	0.55±0.01	0.17±0.00	0.04±0.00
SwBr	28.47±12.63	59.97±9.87	0.85±0.00	3.52±0.34	4.82±0.36	0.05±0.00	0.03±0.00	0.09±0.00	0.44±0.01
TwCe	46.33±5.44	34.68±2.41	1.94±0.00	0.55±0.00	0.01±0.00	0.00±0.00	0.00±0.00	0.00±0.00	0.00±0.00
FwCe	38.38±3.14	47.39±1.99	2.19±0.01	0.96±0.00	0.01±0.00	0.00±0.00	0.00±0.00	0.00±0.00	0.00±0.00
SwCe	75.47±0.79	18.16±0.77	1.48±0.01	1.15±0.01	0.11±0.00	0.00±0.00	0.00±0.00	0.00±0.00	0.00±0.00
TwSp	72.85±4.71	15.16±0.79	1.80±0.03	6.24±0.72	0.61±0.01	0.11±0.00	0.13±0.00	0.05±0.00	0.40±0.01
FwSp	44.51±6.49	45.90±5.19	1.58±0.00	1.76±0.01	0.67±0.02	0.07±0.00	0.06±0.00	0.01±0.00	0.00±0.00
SwSp	24.23±1.58	51.44±3.99	6.17±0.26	5.91±0.07	1.38±0.01	1.11±0.00	0.49±0.00	0.19±0.00	0.05±0.00
TwTu	15.29±1.19	73.25±4.33	1.82±0.01	3.95±0.12	2.16±0.11	0.44±0.00	0.25±0.00	0.56±0.01	0.04±0.00
FwTu	27.92±4.50	31.76±3.86	17.99±7.65	11.60±0.95	1.23±0.01	2.33±0.17	1.60±0.04	0.35±0.00	0.39±0.01
SwTu	63.38±9.9	27.90±5.46	0.88±0.01	3.18±0.16	2.56±0.27	0.23±0.00	0.10±0.00	0.05±0.00	0.25±0.00

Note: Tu, Br, Sp, Ce mean breast tumor, normal breast tissue, spleen tissue and cecal contents respetively; Tw, Fw, Sw mean 3 weeks, 5 weeks and 7 weeks respectively.
