# Supplementary material for: Temporal and Spatial Dynamics of Tumor–Host Microbiota in Breast Cancer Progression
Source: Microorganisms. 2025 Jul 10;13(7):1632. doi: 10.3390/microorganisms13071632 (PMC12300001; doi:10.3390/microorganisms13071632)
Supplement: Supplementary file 1 [file microorganisms-13-01632-s001.zip › Table S2. The abundance of microbiota at the genus level in four tissues during tumor progression.pdf]

Table S2. The abundance of microbiota at the genus level in four tissues (n=5, %)

groups	Streptococcus	Sphingomonas	Lactobacillus	Bifidobacterium	Stenotrophomonas	Bacillus	Bradyrhizobium	Desulfovibrio	Proteus	Helicobacter
TwBr	0.38±0.00	54.29±13.02	7.05±1.50	0.29±0.00	0.61±0.01	0.14±0.00	5.39±0.25	0.60±0.01	0.01±0.00	1.52±0.10
FwBr	1.61±0.02	8.40±0.86	7.70±2.30	10.45±4.93	1.53±0.02	0.50±0.00	14.24±5.38	0.03±0.00	0.04±0.00	0.01±0.00
SwBr	3.70±0.23	22.52±1.86	0.67±0.00	0.10±0.00	0.39±0.00	11.16±6.08	27.84±6.83	0.07±0.00	0.02±0.00	0.04±0.00
TwCe	0.21±0.00	0.01±0.00	41.23±6.21	0.01±0.00	0.00±0.00	0.00±0.00	0.00±0.00	30.39±.89	0.00±0.00	13.48±3.94
FwCe	0.19±0.00	0.05±0.00	25.63±2.39	0.01±0.00	0.01±0.00	0.00±0.00	0.01±0.00	42.80±1.48	0.00±0.00	7.16±0.23
SwCe	0.02±0.00	0.01±0.00	69.48±0.83	0.39±0.01	0.10±0.00	0.00±0.00	0.00±0.00	16.60±0.79	0.04±0.00	2.21±0.02
TwSp	0.16±0.00	2.18±0.03	50.14±19.99	0.66±0.02	3.11±0.18	5.50±1.43	2.79±0.10	3.85±0.26	0.03±0.00	0.62±0.01
FwSp	0.41±0.00	14.65±9.72	25.56±7.58	0.11±0.00	0.42±0.00	0.00±0.00	0.80±0.02	16.55±3.40	9.66±4.65	2.88±0.22
SwSp	1.31±0.01	20.57±4.01	3.38±0.07	0.55±0.00	15.05±7.98	0.32±0.00	3.50±0.16	0.35±0.00	0.05±0.00	0.12±0.00
TwTu	0.31±0.00	51.70±15.14	1.68±0.02	0.55±0.00	1.17±0.01	0.16±0.00	9.81±1.21	0.14±0.00	0.03±0.00	0.22±0.00
FwTu	1.20±0.02	5.48±0.36	2.50±0.14	14.02±8.45	1.62±0.04	0.45±0.00	4.86±0.26	0.05±0.00	0.02±0.00	0.02±0.00
SwTu	20.32±18.23	9.95±0.59	2.81±0.31	0.21±0.00	0.32±0.00	32.95±10.27	12.35±2.46	0.05±0.00	0.01±0.00	0.06±0.00

Note: Tu, Br, Sp, Ce mean breast tumor, normal breast tissue, spleen tissue and cecal contents respetively; Tw, Fw, Sw mean 3 weeks, 5 weeks and 7 weeks respectively.
